# Emerging Topics in Protein Crystallography

**DOI:** 10.3390/ijms25105311

**Published:** 2024-05-13

**Authors:** Zhongzhou Chen, Giuseppe Zanotti

**Affiliations:** 1 State Key Laboratory of Animal Biotech Breeding and Beijing Advanced Innovation Center for Food Nutrition and Human Health, College of Biological Sciences, China Agricultural University, Beijing 100193, China; 2 Department of Biomedical Sciences, University of Padova, Via Ugo Bassi 48B, 35131 Padova, Italy

Protein crystallography is the discipline concerned with the determination of the three-dimensional structure of biological macromolecules in a crystalline state. It took its first steps in the 1950s [1], and today, we can confidently assert that it has reached a phase of complete development [2]. Technically, bio-crystallography has undergone an extraordinary evolution in its more than seventy years of existence, but it could not have made the grand strides that have led it to be a technically mature discipline without the tumultuous parallel development of computing systems and related software [3,4,5,6]. Much water has passed under the bridge since computers were the size of cabinets that occupied numerous rooms and received their input data through punched cards; today’s tools are a little larger than a tablet and have computing and data storage power unimaginable at the beginning. Crystallography has not only benefited from the developments of computer tools, both hardware and software, but it has also been strongly aided by the development of X-ray sources, particularly synchrotrons [7], X-ray detectors [8], and the improvement in crystallization methods (contribution 1). To give an immediate idea of the progress in the field, we recall that at the time of its foundation, the Protein Data Bank (PDB, http://www.rcsb.org, accessed on 9 May 2024), a database in which the coordinates and data of all resolved three-dimensional structures of macromolecules in the world are deposited, contained 12 structures. Today this number has exceeded 218,000, the vast majority of which (more than 84%) were determined using X-ray diffraction [9].

At this point, we must mention the development of what is defined as cryo-Electron Microscopy (cryo-EM), a technique that allows the determination of the three-dimensional structure of biological macromolecules at nearly atomic level from an ultra-thin frozen layer of the protein solution, without the need for the protein to be present in crystalline form [10,11,12]. This, in addition to the absence of the phase problem, has opened up the field to the determination of structures of huge macromolecular complexes, almost impossible to obtain in crystalline form and generally present in very small quantities within the cell.

In this context, it is understandable that this Special Issue received only five contributions, of which only two are of a methodological nature. We can confidently state that much of the technology inherent to the crystallographic technique has already been extensively developed in recent decades, and there are few truly significant developments on the horizon. We might expect more in terms of new insights into the structure of macromolecules particularly relevant in the field of human health, although, in this area, we cannot ignore the contribution of artificial intelligence, which has allowed the building of a database containing the prediction of the three-dimensional structures of essentially all of the proteins of numerous genomes [13,14]. Although these are predictions, they appear to be quite reliable and are often sufficient to draw conclusions about the functional and mechanistic aspects of the biological process. The only aspect where predictions are still particularly lacking concerns macromolecular complexes and protein–small-molecule complexes, although major progress is expected in this field in the coming years.

The two methodological articles presented in this Special Issue concern improvements in the structure determination process. Carrozzini et al. (contribution 2) describe REMO22, a software program that uses prior information to bypass the phase problem using a computational technique called molecular replacement, which involves using an existing or predicted molecular model to determine a new structure based on an approximate existing model (and, of course, the crystal diffraction data of the structure to be determined). REMO22 represents an evolution of the previous software REMO09 and is inserted into a pipeline that also includes additional computational stages, such as phase refinement and automated model building. This pipeline tends to be completely automatic for solving the structures of proteins and nucleic acids, with minimal intervention from the operator. In the other methodological work, Ma et al. (contribution 3) describe the use of an anomalous signal for the correct positioning and orientation of protein fragments containing anomalous scatterers. The technique has been used, in particular, for positioning in the electron density of fragments containing chlorine and sulfur in the non-structural protein 1 (nsp1) of the most recent dangerous pandemic SARS-CoV-2, but the method is generally applicable for any anomalous scatterers. Its use requires a tunable source that can reach the appropriate wavelengths, possibly at low energy in vacuum. In the case in question, the authors, by measuring data at two different energies, were able to identify multiple orientations in various fragments containing chlorine or sulfur.

The other three papers in the Special Issue are of an applied nature. Gao et al. (contribution 4) describe the structural characterization of the enzyme N-Acetyl Sugar Amidotransferase, an enzyme involved in the biosynthesis of lipopolysaccharides in the bacterium *Legionella pneumophila*. The structure, resolved at 2.33 Angstrom resolution, contains a Rossmann-like fold with a PP loop, suggesting that the catalyzed reaction probably requires the conversion of ATP to AMP and PPi. In their work, Shang et al. (contribution 5) solve three complex structures of 6 mA demethylase *Caenorhabditis elegans* NMAD-1A through rational mutations and find a unique “stretch-out” conformation of its DNA binding region. Biochemical and structural studies reveal that it preferentially demethylates 6 mA, an emerging epigenetic mark, in the unpairing regions and binds to the nucleosome with the help of the carboxy-terminal domain and the zinc finger domain.

The last paper, Del Giudice et al. (contribution 6), is not strictly crystallographic, but describes the use of the SAXS (Small-Angle X-ray Scattering) technique in solution to determine the content of disordered structure in the different redox states of the enzyme CP12 from *Arabidopsis Thaliana*, a protein universally distributed in all photosynthetic organisms and involved in regulating the Calvin–Benson cycle of photosynthesis. Considering different levels of disorder, the SAXS data suggest that the reduced form is completely disordered, and that the oxidized form is better described by conformers that include partially ordered portions in some areas, particularly around the sulfide bridge, which coexist with portions that remain disordered.
